# Spectral Efficiency Augmentation in Uplink Massive MIMO Systems by Increasing Transmit Power and Uniform Linear Array Gain

**DOI:** 10.3390/s20174982

**Published:** 2020-09-02

**Authors:** Jehangir Arshad, Abdul Rehman, Ateeq Ur Rehman, Rehmat Ullah, Seong Oun Hwang

**Affiliations:** 1 Electrical and Computer Engineering Department, COMSATS University Islamabad, Lahore Campus, Punjab 54000, Pakistan; jehangirarshad@cuilahore.edu.pk; 2 Department of Computer Science and Engineering, Kyungpook National University, Daegu 41566, Korea; a.rehman.knu@knu.ac.kr; 3 College of Internet of Things Engineering, Hohai University, Changzhou 213022, China; ateeq@hhu.edu.cn; 4 Department of Computer Engineering, College of IT Convergence, Gachon University, Seongnam 13120, Korea

**Keywords:** transmit power, line-of-site, non-line-of-site, channel gain, future networks, spectral efficiency, area throughput, uniform linear array, signal-to-noise ratio, signal-to-noise interference ratio, inter-cell interference

## Abstract

Improved Spectral Efficiency (SE) is a prominent feature of Massive Multiple-Input and Multiple-Output systems. These systems are prepared with antenna clusters at receiver $\left(R_{x}\right)$ and transmitter $\left(T_{x}\right)$. In this paper, we examined a massive MIMO system to increase SE in each cell that ultimately improves the area throughput of the system. We are aiming to find appropriate values of average cell-density (D), available bandwidth (B), and SE to maximize area throughput because it is the function of these parameters. Likewise, a SE augmentation model was developed to attain an increased transmit power and antenna array gain. The proposed model also considers the inter-user interference from neighboring cells along with incident angles of desired and interfering users. Moreover, simulation results validate the proposed model that is implementable in real-time scenarios by realizing maximum SE of 12.79 bits/s/Hz in Line of Sight (LoS) and 12.69 bits/s/Hz in Non-Line of Sight (NLoS) scenarios, respectively. The proposed results also substantiate the SE augmentation because it is a linear function of transmit power and array gain while using the Uniform Linear Array (ULA) configuration. The findings of this work ensure the efficient transmission of information in future networks.

## 1. Introduction

An exponential increase in mobile phone users and the inclusion of smart gadgets in daily-life affairs has overburdened the cellular networks. Quality-of-service, high data rate, energy efficiency, remote connectivity, and increased network capacity at affordable costs are the major requirements of future networks. The wireless communication technology has significantly changed the methods of information interchange. The use of satellites has provided liberty with wireless access to remote locations. Additionally, Wi-Fi-based Local Area Networks (LANs) and UMTS2, GSM1, and LTE3 based cellular Wide Area Networks (WANs) improved this area in all demanded aspects. Recently, wireless connectivity has been accepted as a basic necessity of society because of an exponential increase in services and applications. According to Martin cooper’s law [1], the number of connections (both data and voice) doubled every 2.5 years. Moreover, Ericsson Mobility verifies a composite 12-monthly growth rate of 42% in transportable data traffic from 2016–2022 [2] that is even quicker than the prediction made in [1]. An imperative query for researchers is, how does one develop existing and/or new technologies to meet the increasing requirements, and thus evade the crisis of data traffic? The end-users expect wireless connectivity services at any place and at any time. The pervasive connectivity and exponential traffic growth urge the researchers to plan groundbreaking wireless technologies.

**Figure 1:**
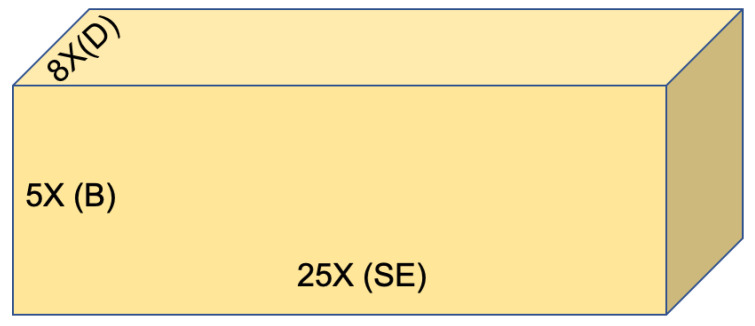
Rectangular container with coordinates of D,B,andSE.

This paper provides an examination of massive MIMO technology to validate how and why it is a proficient solution to knob extra data traffic than existing wireless technology. The prominent aim of this work is to select appropriate values of *B*, *D*, and SE to optimize the area throughput with 1000x as shown in Figure 1. A realistic method is to examine an appropriate value of SE that can be used together with increasing D and B to realize 1000x goal. Mobile networks were initially intended for voice communications; however, currently, data transmission has dominated [3,4]. Furthermore, video streaming is considered to be a key driver of the forecasted rise in data traffic demand [5]. The area throughput is thus an extremely related performance parameter of modern wireless networks that is measured in bits/s/km${}^{2}$ and modeled as Equation (1).

(1) $$AreaThroughput=D.B.SE\;{\mathrm{Bits}/s/\mathrm{km}}^{2}=\mathrm{Hz}.\;{\mathrm{cells}/\mathrm{km}}^{2}.\;\mathrm{bit}/s/\mathrm{Hz}/\mathrm{cell}$$

**Figure 2:**
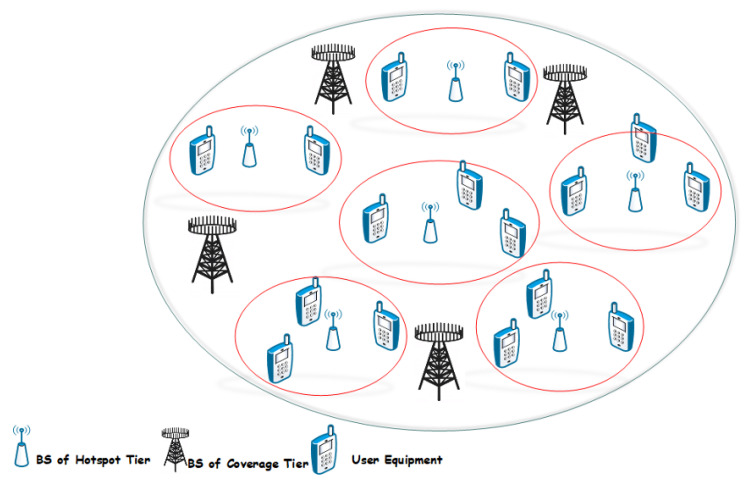
Illustration of hotspot tier and coverage tier.

The SE can be further defined as “total information transferred in one second by using 1 Hz bandwidth”. In (1), parameters D,B, and SE are three key parameters to optimize the area throughput in a massive MIMO technology for future networks. In coverage prospects, a wireless network can be divided into two tiers described in Figure 2 the coverage tier and hotspot tier. The definition of area throughput can be considered a principle for both tiers. The area throughput can be considered to be a volume of a rectangular container with coordinates of D,B, and SE [6].

The parameters shown in Figure 1 are dependent on each other as choosing cell density and frequency band influences broadcast environments. All three parameters can be treated independently for the 1st order approximation. This query can be settled by increasing bandwidth up to 1000-fold. Existing networks use approximately 1 GHz bandwidth i.e., in Sweden, mobile phone operators can use a 1 GHz spectrum, while approximately 650 MHz in the United States with a supplementary 500 MHz available for Wi-Fi [7,8]. A network intended with 1000-fold improvement would approximately use 1 THz that is unrealistic. Additionally, the frequency spectrum is a global resource used for different services, and it needs higher frequency bands that physically restrict the service range reliability.

The second option would be the densification of the network by deploying 1000x BS/km${}^{2}$. In existing deployment scenarios, the distance between BSs is a few 100 m in the coverage tier, in which BSs are positioned at raising sites to circumvent from shadowing of huge buildings and objects. The scenario provided in Figure 2 gives an illustration of the hotspot tier and coverage tier. It confines several sites for the deployment of BSs in coverage tier. Additionally, BS densification would be challenging unless the BSs are moved closer to User Equipment (UEs) that increases the risk of deep shadowing, in that way plummeting the coverage. However, the deployment of extra hotspots is comparatively a more feasible solution. The distance between BSs (in hotspot tier) can surely be reduced to a few meters in future network deployments. Even underneath much densification in hotspot tier, coverage tier still needs to duck coverage holes and provide mobility support. The technique for area throughput optimization is to optimize SE in future mobile networks. It is predominantly significant for BSs that can neither depend upon network densification nor uses mm-Wave band.

Furthermore, optimization of SE corresponds to use bandwidth and BSs, which are efficiently placed by using new multiplexing and modulation methods. Modulation and channel coding play a crucial part in the physical layer to enhance SE. Essentially, higher SE can be attained by implementing a higher-order modulation scheme and low-code rate with high SNR. In [9], the authors have developed a novel approach to improve bit-error-rate (BER) performance of iterative detection and decoding (IDD) schemes by using a Low-Density Parity Check (LDPC) codes. Recently, a novel family of protograph LDPC codes also called Root-Protograph (RP-LDPC) codes are used in [10]. The presented codes can realize highspeed decoding and encoding by quasi-cyclic structure. It can also achieve near-outage-limit performance in Block-Fading (BF) set-ups [11,12].

Last but not least, another aspect of SE augmentation in massive MIMO systems and antenna array elements is a mutual coupling. If mutual coupling increases it drastically affects the antenna characteristics by degrading the system’s performance [13]. A lot of existing works presented novel way outs of reducing mutual coupling specifically, patch antennas using UC-EBG superstrate [14], closely spaced microstrip MIMO antennas [15,16], mutual coupling in closed packed antennas [17], and micro coupling in planner antennas by using a Simple Microstrip U-Section [18]. The mutual coupling between closely packed antennas rises either by the large flow of surface current from the exciting ports or space radiation and surface waves. Additionally, the opposing effect of mutual coupling on reflection coefficients cannot be undervalued [19]. Hence, limiting the mutual coupling is a challenging task within the recent miniaturized printed and other antennas in designing of massive MIMO antenna systems. In digital MIMO infrastructure, the higher mutual coupling effects error rate and channel capacity. An extensive range of coded modulation schemes is proposed to decrease this effect, such as partial swam optimization, genetic algorithms, and galaxy-based search algorithms.

### Preliminaries

The 1000x area throughput is accomplished without using mm-Wave spectrum and/or any extensive densification since it would unavoidably result as a patchy in the coverage tier. To avoid pitchy coverage, improved SE is desired. In this work, we have established an argument that the massive MIMO is capable of providing enhanced SE. Contrarily, the hotspot tier reduces burden of coverage tier by unburdening a huge share of traffic from low mobility user equipment. Subsequently, hotspot tier has been boosted with cell-densification and by hefty bandwidth accessible in mm-Wave. The Shannon proposition of sampling infers that ‘the band-limited data communication signal transmitted through a channel with bandwidth ‘B’ can be completely recovered by ‘2B’ equal spaced and real value samples/s [11] While considering the complex baseband signal, B complex-valued samples/s is in natural quantity [12]. These samples are the degrees of freedom (DoF) offered to construct a communication signal. The SE is amount of information transferred reliably per complex-valued sample. For a fading channel between UE and BS, SE is the number of information bits transmitted reliably over communication channel measured as bits/s/Hz. Moreover, an information rate is the product of SE and B which is another associated metric measured in bit/s. For all channels from UEs to their particular BS in a cell, sum SE is measured in bit/s/Hz/cell. The channel between a Tx and Rx at specified locations can serve several UEs with respect to the used encoding and decoding scheme. According to Shannon’s channel capacity [20,21,22], the max. SE can be calculated by channel capacity that is demonstrated in Equation (2). Suppose, a communication channel with input and output are represented by random variables a and b, respectively. The channel capacity (C) can be calculated as Equation (2) by taking the supremum concerning all possible f(x) input distributions.

(2) $$C=Sup_{f(x)}H\left(b\right)-H\left(b|a\right)$$

whereas the H(b) and H(b∥a) represents the differential and conditional-differential entropies of the *b* given the *a*. The channel capacity in Equation (2) can be calculated as in Equation (3) [11].

where $n=N_{c}$$\left(0,\sigma^{2}\right)$ is independent noise, $E\{{\left|a\right|}^{2}\}\leq p$ gives the power-limited input distribution and the *x* describes the channel response $\left(x\in C\right)$ that is a known value. The ergodic channel capacity can be attained as Equation (3) by input $a∼N_{C}\left(0,p\right)$.

(3) $$C=E\left\{\log_{2}\left(1+\frac{p{\left|x\right|}^{2}}{\sigma^{2}}\right)\right\}$$

In Equation (3), $p{\left|x\right|}^{2}/\sigma^{2}$ is an actual compute-able SNR for a channel response (x), where it is an instantaneous SNR for a specified channel realization with random value of channel response. From Equation (3), the average SNR has been defined as $pE{\left|x\right|}^{2}/\sigma^{2}$ while $E{\left|x\right|}^{2}$ is an average channel gain and expectation has been calculated according to the channel realizations. In wireless networks, the information signals tainted by interference occurred in the same and other cells. This interference is modeled at the output of a memory-less channel. The interference is reliant on input and channel response and it is challenging to realize the precise channel capacity of interference channels; however, expedient lower-bounds are calculated. By using [23,24,25,26], the lower-bound capacity of a channel with input and output calculated as Equation (4).

If *x* is deterministic and the interference *y* has mean equals to zero, a known value of variance $p_{y}\in R_{+}$ and uncorrelated input (i.e., E{a∗y}=0), in this way the lower-bounded channel capacity can be calculated as Equation (4)

(4) $$C\geq \log_{2}\left(1+\frac{p{\left|x\right|}^{2}}{p_{y}+\sigma^{2}}\right)$$

while the bound is realized employing $a=N_{C}\left(0,p\right)$. Suppose *x* as an alternative is a realization of a random variable and that is random variable by *r*’ the realization that disturbs the interference variance. If *n* is independent of *y* given *x* and *r*, mean equals to $E\left\{y|x,r=0\right\}$ and variance is $p_{y}\left(x,r\right)=E\left\{{\left|y\right|}^{2}|x,v\right\}$. Hence, the interference is uncorrelated with the given input $\left(i.e.,\;E\{a*y|x,r\}=0\right)$ and lower-bound ergodic capacity can be determined as Equation (5)

(5) $$C\geq E\left\{\log_{2}\left(1+\frac{p{\left|x\right|}^{2}}{p_{y}+\sigma^{2}}\right)\right\}$$

The capacity attained in Equation (5) is accomplished by less complex signal processing at the receiver, in which the interference is considered to be noise. Moreover, the Signal to Interference Noise Ratio (SINR) can be given as Equation (6)

(6) $$SINR=\frac{p{\left|x\right|}^{2}}{\sigma^{2}+p_{y}}$$

## 2. System Model and Proposed Methods to Enhance Se

**Figure 3:**
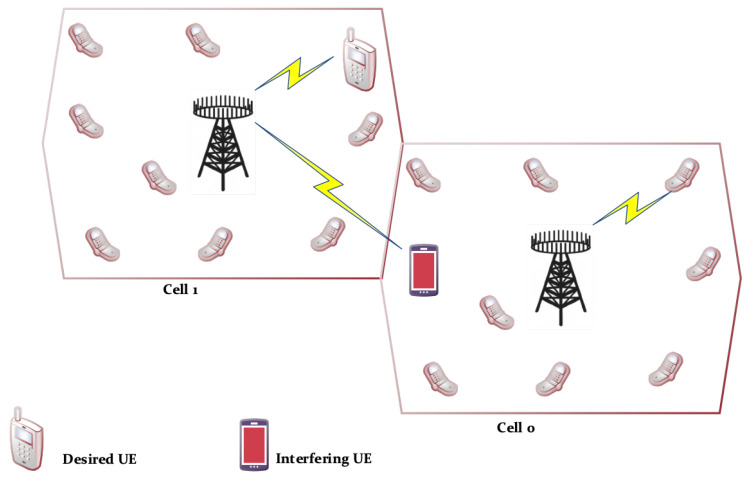
A Two Cell Scenario: Channel gain between every UE and BS is identical in each cell.

The SE is improved using different methods. To keep it simple, a 2-cell network was considered, in which the typical channel gain between every UE and BS is identical in each cell, as shown in Figure 3. It is a docile system model to study the fundamental characteristics of wireless communication networks a smaller number of cells means a smaller number of parameters to deal with. It is illustration of Wyner model [27,28] for fading channels. In the up-link (UL) scenario shown in Figure 3, the UEs in cell 0 transmit data to their corresponding BS, where the UL communication signals of cell 1 UEs interferes with the UEs of cell 0. Table 1 represents the symbols and their description used in the proposed signal model. The avg. channel gains are taken as very smaller ranging from −70 dB to −120 dB vales because the energy of communication signal decays as it passes through the propagation environment. For ease, we are inspired by the supposition made in [26,27], $g_{0}=g_{1}$ and $y_{0}=y_{1}$, hence $\bar{g}=y_{0}/g_{0}=y_{1}/g_{0}=y_{0}/g_{1}=y_{1}/g_{1}$.

### 2.1. Increase the Transmit Power

**Figure 4:**
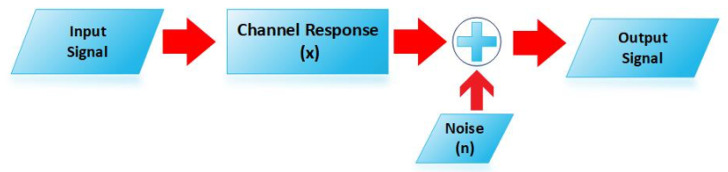
Desired signal, interference from other cell and noise added to the signal during transmission.

The SE of a cellular network is certainly dependent on the value of avg. Signal to Noise Ratio defined as $pE{\left|x\right|}^{2}/\sigma^{2}$. By using Wyner’s model, the avg. SNR of a UE in cell $0^{\mathrm{th}}$ is represented by $SNR_{0}$ can be molded as $\left(\frac{p}{\sigma^{2}}g_{0}\right)$ where *p* and $\sigma^{2}$ represents the transmit power of UE and noise power, respectively measured in Joule/time interval. The symbol sampled complex base band signal $\left(m_{0}\in C\right)$ received at the BS in cell 0 is the sum of the desired signal, interference from other cell and noised added to the signal during transmission that can be represented as Figure 4 and also in Equation (7):

(7) $$m_{0}=x_{0}I_{0}+x_{1}I_{1}+n_{0}$$

whereas the $n_{0}$ represents the demonstrated as $n∼N_{c}$$\left(0,\sigma^{2}\right)$. The scalar quantities $I_{0}$ and $I_{1}$ are the information symbols transmitted from interfering and desired UEs where $I_{0},I_{1}∼N_{C}\left(0,p\right)$. Furthermore, the channel responses of $I_{0}$ and $I_{1}$ are designated by $x_{0}$ and $x_{1}$$\left(x_{0},x_{1}\in C\right)$.

The channel response properties are contingent on the broadcast environment hence, we consider a model for Line-of-Sight (LoS) for which $x_{0}$ and $x_{1}$ are deterministic scalars corresponding to the square root of average channel gains modeled as $x_{i}=\sqrt{g_{i}}$ for i=0,1 and the other one for non-Line-of-Sight (NLoS) propagation.

Generally, channel response also includes the phase rotation; however it has been ignored here as the SE is not affected by this. In LoS, $g_{i}$ would be taken as a microscopic large-scale fading, instigated due to distance related path loss components. The transceiver hardware impact and antenna gain are also engrossed in this parameter. Moreover, it has been considered a constant if receiver and transmitter are stationary, although it is variable if receiver and /or transmitter move. The prescribed microscopic movement has been represented by $x_{i}$ and further modeled as phase rotations. For the deterministic channels, $x_{i}$ is considered to be a constant to apply the SE modeled in Equation (5). In NLoS milieus, channel responses are random variables that variate over frequency and time. If scattering among BS and UE is sufficient, $x_{0}$ and $x_{1}$ modeled as $x_{i}∼N_{C}\left(0,g_{i}\right)$ where i=0,1 given in [29,30,31,32]. The receiver receives the signals arriving from different paths and the overlaid signals can either cancel or reinforce. In the case of a large number of paths, Gaussian distribution was used with a central limit theorem also known as small scale-fading. Contrarily, the variance $g_{i}$ is microscopic large-scale fading that contains shadowing, path loss component, penetration loss and antenna gains in non-LoS propagation environment. In $x_{i}∼N_{C}\left(0,g_{i}\right)$, the channel model is Rayleigh fading channel as $\left|x_{i}\right|$ is a random variable with Rayleigh distribution. Additionally, an avg. channel gain is $E\left\{{\left|x_{i}\right|}^{2}\right\}=g_{i}$, for I=0,1, in LOS and non-LoS propagation so that both are easily compared. A closed-form up-link SE for anticipated UE (for both LoS and non-LoS) can be modeled as in Equations (8) and (9).

(8) $$Spectral\;Efficiency_{LoS}^{0}=SE_{LoS}^{0}=\log_{2}\left(1+\frac{1}{\bar{g}+\frac{1}{SNR_{0}}}\right)$$

where the $SNR_{0}$ and $\bar{g}$ can be calculated as $\frac{p}{\sigma^{2}}$$g_{0}$ and $\frac{y_{0}}{g_{0}}=\frac{y_{1}}{g_{0}}=\frac{y_{0}}{g_{1}}=\frac{y_{1}}{g_{1}}$, respectively. To keep it simple, suppose $v=\frac{1}{\bar{g}*SNR_{0}};u=\frac{1}{SNR_{0}}$.

(9) $$\begin{array}{c} Spectral\;Efficiency_{NLoS}^{0}=SE_{NLoS}^{0}=\log_{2}\left(1+\frac{p{\left|x_{0}\right|}^{2}}{p{\left|x_{1}\right|}^{2}+\sigma^{2}}\right)=\frac{e^{u}E_{1}\left(u\right)-e^{v}E_{1}\left(v\right)}{\log_{e}2\left(1-\bar{g}\right)} \end{array}$$

$E_{1}\left(x\right)=\int_{1}^{\infty }\frac{e^{xl}}{l}$dl and $\log_{e}\left(\;\cdot \;\right)$ symbolizes the exponential integral and natural logarithm, respectively. The SE is certainly an increasing function of SNR that can be seen from (11), whereas it is a logarithm SINR modeled in (10).

(10) $$SINR=\bar{g}+\frac{1}{SNR_{0}}=\frac{signal\;power}{Interference\;Power+Noise\;Power}=\frac{pg_{0}}{py_{0}+\sigma^{2}}$$

The SE can be increased by increasing transmit power *p* of the signal that can be modeled as Equations (11) and (12) for LoS and non-LoS.

(11) $$SE_{LoS}^{0}=\log_{2}\left(1+\frac{1}{\bar{g}}\right)\;p\to \infty$$

whereas the limit is computed according to the interference strength. Moreover, the corresponding limit NLoS limit can be modeled as Equation (12)

(12) $$SE_{NLoS}^{0}=\frac{1}{1-\bar{g}}\log_{2}\left(\frac{1}{\bar{g}}\right)\;p\to \infty$$

### 2.2. Enhanced Se by Enhancing Array Gain

**Figure 5:**
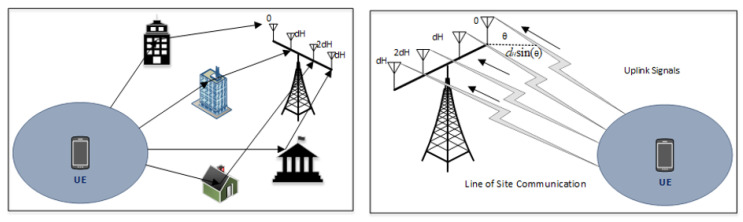
UL communication scenarios for LoS and NLoS signals describing arrival angles.

As an alternative to an increase in transmit power (UL), multiple base station antennas deployed to amass extra energy from electromagnetic waves. This multiple antenna deployment at BS also known as “adaptive or smart” uses a spatial filtering scheme that permits the receiver to differentiate different spatial directivity signals [33,34,35]. Again, in this method, we will keep simplicity in mind and consider the same 2 cell scenarios to develop an understanding as shown in Figure 3. In the $0^{\mathrm{th}}$ cell shown in Figure 3, an array of *N* antennas is deployed at BS and the channel responses are represented by $x_{0},x_{1}\in C^{N}$ from the desired UE and the interfering UEs, respectively. The channel response of the $n^{\mathrm{th}}$ element for each vector can be detected at $n^{\mathrm{th}}$ antenna at BS for n=1,2,3,..,N. The received UL scalar signal as in Equation (7) is further protracted to calculate $x_{0}$ while $n_{0}∼N_{C}\left(0_{N},\sigma^{2}I_{N}\right)$ represents the noise vector received at antenna array and $I_{0}$ and $I_{1}$ representing the information symbols similar as provided in Equation (7). We have used horizontal-uniform linear array with $d_{H}$ antenna spacing from 0 to 0.5, wavelength λ at carrier frequency for LoS case, hence, the spacing of antennas can be calculated in meters as $\lambda d_{H}$. All user locations are fixed that provides deterministic channel response $\left(x_{i}\right)$ as Equation (13) [36,37].

(13) $$x_{i}=\sqrt{g_{i}}{\left[e^{2\pi jd_{H}sin\left(\theta_{i}\right)}\;\cdot \;\;\cdot \;\;\cdot \;e^{2\pi jd_{H}\left(N-1\right)sin\left(\theta_{i}\right)}\right]}^{T}for\;i=0,1$$

where $\theta_{i}$ is an azimuth angle to UE w.r.t the BS array bore sight in $0^{\mathrm{th}}$ cell ranging from $\left[0,2\pi \right]$, and $g_{i}$ represents the large-scale fading coefficient. The $x_{i}$ in (19) is ignored as it has no effect on SE modeling. The UL LoS model of signal propagation has been demonstrated in Figure 5, where a plane EM wave is reaching the antenna arrays with an azimuth angle represented by θ. In Figure 2, a comparison of 2 inline adjacent antennas are shown, one signal traveled a distance of $d_{H}sin\left(\theta \right)$ lengthier than the other signal. It gives an array response given in Equation (13) with phase rotations multiple of $d_{H}sin\left(\theta \right)$.

In Figure 5, a scattered NLoS environment isn presented, for which channel response is considered spatially uncorrelated. Hence, $x_{i}∼N_{C}\left(0_{N},g_{i}I_{N}\right)$ for i=0,1 according to cell 0 and 1. Whereas the $g_{i}$ labeled as a large-scale fading coefficient. Additionally, the Gaussian distribution and randomness account for $g_{i}$. The channel in Figure 5 is Independent & Identically Distributed (I.I.D.) Rayleigh fading or uncorrelated Rayleigh fading because of $x_{i}$ elements are uncorrelated/independent and have Rayleigh distributed magnitudes. This channel model is tractable for highly scattered environments, where a BS array is fenced by many scattering objects i.e., buildings. The benefits of massive antennas at BS are taken if channel response from desired user is known to BS that allows it to combine received signals coherently arrived from all other antennas. For this task, it is assumed that BS knows channel responses and used to choose a receive-combining vector represented by $w_{0}$ where $w_{0}\in C^{N}$. The $w_{0}$ is multiplied with received signal as Equation (14).

(14) $$w_{0}^{H}m_{0}=w_{0}^{H}\left(x_{0}I_{0}+x_{1}I_{1}+n_{0}\right)$$

There are different received combining methods; however, maximum ratio (MR) combining shows promising results in the existing literature, and is defined as $w_{0}=x_{0}$. It provides maximum ratio calculated as ${\left|x_{0}w_{0}^{H}\right|}^{2}/{∥w_{0}∥}^{2}$. Supposing the BS of $0^{\mathrm{th}}$ identifies the channel responses to apply MR combining on the signal calculated in Equation (14). The achievable up-link spectral efficiency for the desired user (LoS case) is modeled as Equation (15)

(15) $$Spectral\;Efficiency_{LoS}^{0}=SE_{LoS}^{0}=\log_{2}\left(1+\frac{N}{\bar{g}g\left(\theta_{0},\theta_{1}\right)+u}\right)\;p\to \infty$$

where *u* is $1/SNR_{0}$, and g(ϕ,ψ) function is calculated as Equation (16)

(16) $$g\left(\theta ,\phi \right)=\left\{\begin{array}{cc} \frac{sin^{2}\left(\pi d_{H}N\left(sin\left(\theta \right)-sin\left(\phi \right)\right)\right)}{Nsin^{2}\left(\pi d_{H}N\left(sin\left(\theta \right)-sin\left(\phi \right)\right)\right)} & \mathrm{if}\;sin(\theta )\neq sin(\phi )\\ N & \mathrm{if}\;sin(\theta )=sin(\phi ) \end{array}\right.$$

Likewise, achievable uplink spectral efficiency for the desired user (NLoS case) is modeled as Equation (15) with $\bar{g}=1$.

(17) $$\begin{array}{c} SE_{NLoS}^{0}=\log_{2}\left(-1+\frac{1}{1-{\frac{1}{\bar{g}}}^{N}}\right)\frac{e^{v}E_{1}\left(v\right)}{\log_{e}2}\\ +\sum_{n=1}^{N}\sum_{L}^{N-n}\frac{{\left(-1\right)}^{N}-n-L+1\left(e^{u}E_{1}\left(u\right)+\sum_{z=1}^{L}\frac{1}{z}\sum_{j=0}z-1\frac{1}{j!SNR_{0}}\right)}{{\left(1-\frac{1}{\bar{g}}\right)}^{N}\left(N-n-L\right)!SNR_{0}\bar{g}\log_{e}2} \end{array}$$

where $E_{1}\left(x\right)=\int_{1}^{\infty }\frac{e^{xl}}{l}$ represents tan exponential integral and n! signifies the factorial function. In Equation (16) and (17), it can be observed that the SE is branded by the desired signal’s SNR, $SNR_{0}$, $\bar{g}$, inter-cell interference strength and *N*. Upper bound of interference-power $\bar{g}g\left(\theta_{0},\theta_{1}\right)$ given in Equation (15) can be calculated as Equation (18)

(18) $$\bar{g}g\left(\theta_{0},\theta_{1}\right)\leq \frac{\bar{g}}{N}\frac{1}{Nsin^{2}\left(\pi d_{H}N\left(sin\left(\theta_{0}\right)-sin\left(\theta_{1}\right)\right)\right)}$$

where $\left(sin\left(\theta_{0}\right)\neq sin\left(\theta_{1}\right)\right)$ that declines to 1/N if additional receiver antennas are deployed. The desired and interfering signals gauge linearly with *N* as both signals reach an identical angle. Practically, it never occurs, however from Equation (18) it can be inferring that the interference is stouter if the arrival angles of both signals are the same. We can use sin(πs)≈πs for $\left|s\right|<0.2$ to demonstrate as in Equation (19)

(19) $$g\left(\theta ,\phi \right)=\frac{sin^{2}\left(\pi d_{H}N\left(sin\left(\theta \right)-sin\left(\phi \right)\right)\right)}{Nsin^{2}\left(\pi d_{H}N\left(sin\left(\theta \right)-sin\left(\phi \right)\right)\right)}\approx \frac{{\left(sin^{2}\left(\pi d_{H}N\left(sin\left(\theta \right)-sin\left(\phi \right)\right)\right)\right)}^{2}}{\left(Nsin^{2}{\left(\pi d_{H}N\left(sin\left(\theta \right)-sin\left(\phi \right)\right)\right)}^{2}\right)}=N$$

whereas $\pi d_{H}N\left(sin\left(\theta \right)-sin\left(\phi \right)\right)<0.2$. The angular-interval turn out to be smaller as $d_{H}N$ of Uniform Linear Array (ULA) rises, however it occurs for any finite size antenna array. Moreover, it is determined that $d_{H}N$ that regulates the angular resolution, in which the interference is abridged by either increasing *N* and/or extending an $d_{H}$(antenna spacing).

## 3. Results And Discussion

This section provides the details of the simulation setup and results of previously discussed methods to increase SE. We have considered a 2-cell scenario for simulation to keep it simple, in which the typical channel gain between every UE and BS is identical in each cell. Moreover, Monte Carlo realizations of the Rayleigh fading has been considered. Table 2 provides the list of simulation parameters.

**Figure 6:**
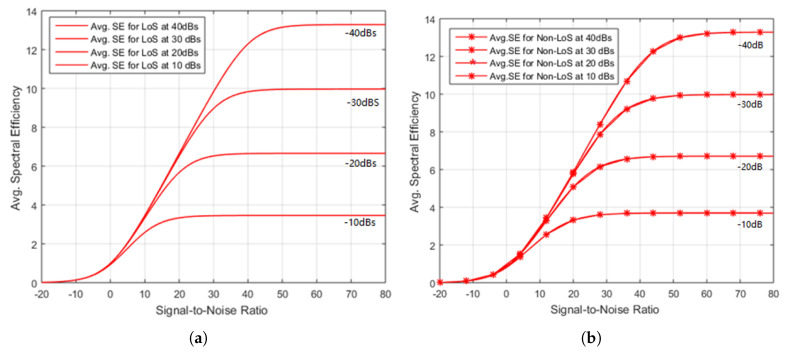
(**a**) Illustration of results for LOS by Increasing Transmit Power, (**b**) Illustration of results for NLoS by Increasing Transmit Power.

Figure 6 shows the results for LoS and NLoS signal arrival in which, the spectral efficiency has been plotted against the increasing values of signal to noise ratio. According to the plot, the SNR is taken as a transmit power *p*. In the simulation, the interference among cells have been represented by $\bar{g}\in \left[-10,-20,-30,-40\;\mathrm{dB}\right]$. In Figure 6a,b, SE for both LoS and NLoS is calculated against the SNR as modeled in Section 3. Figure 6a illustrates the results for LoS at interference of −10 dBs, 20 dBs, −30 dBs and −40 dBs. The SE approaches to its maximum converge quickly that is around 3.8 bit/s/Hz at −10 dBs. The NLoS with similar SNR of −10 dB in Figure 6b reaches its limit value 3.7 bit/s/Hz. For LoS at 40 dBs, the SE approaches to its maximum converge slowly that is around 12.79 bit/s/Hz and the NLoS reaches its limit value 12.79 bit/s/Hz. It has been noticed from the following figure that the increasing $SNR_{0}$ from 20 dB to 40 dB increases the SE with the same ratio. It is also observed that LoS provides slightly higher SE as compared to NLoS for most values of SNR due to the haphazard changes channel response value ${\left|x_{0}\right|}^{2}$.

Nevertheless, at higher values of SNR, the NLoS provides slightly better results since the interference is frailer as compared to the desired signal. It happens because the interference signal cannot be separated from the desired signal in one reflection. In existing networks, this is known as an interference-limited regime, in which the coverage tier operates.

**Figure 7:**
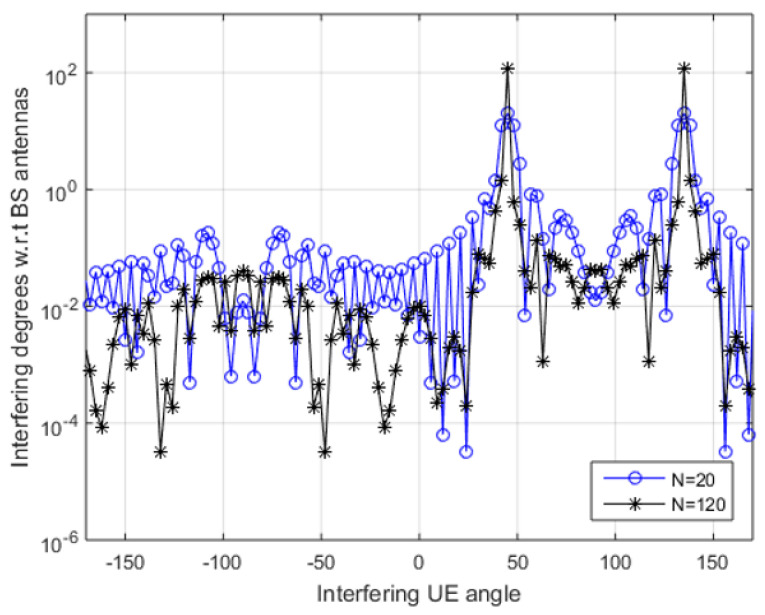
Desired signal, interference from other cell and noise added to the signal during transmission.

Figure 6b presents the spectral efficiency vs SNR of the proposed scheme. If we compare the results of Figure 6b with the results presented in [38] in which, the authors have used an ideal adaptive detector for different SNR and SIR scenarios. A significant improvement can be observed in our proposed results and results of [38]. According to Figure 6b, the proposed scheme shows around 12.7 bits/s/Hz of SE by considering a multicell scenario while modeling inter-cell and inter-user interferences. However, the authors in [38] have considered only one cell scenario that misses the interference factor from other cells and the maximum achieved value is around 8.5 bits/s/Hz. The proposed SE augmentation method shows around a 25% increase in comparison with existing work. Moreover, while we are considering IUI and ICI interferences, we have also modeled the incident and interfering angles of interfering and desired users presented in Figure 7. That is not provided in the existing literature.

The range of spectral efficiency given in Figure 6b can be compared with [39,40,41,42], in which a temporary network deployed that delivers 0 to 5 bits/s/Hz in similar values of interference. Conclusively, it was observed a simple approach for power scaling is not appropriate to realize optimized SE. The interfering degrees concerning BS antennas or $\bar{g}g\left(\theta_{0},\theta_{1}\right)$ is plotted in Figure 7, in which $\theta_{0}$ for desired UE has been fixed at $45^{\circ }$ and $\theta_{1}$ for interfering UE varies from ±180 degrees where $d_{H}$ is half of the wavelength. In case of single antenna, $g(\theta_{0},\theta_{1})$ is 1 regardless of incident angles of signals.

Figure 7 shows the interference peaks when the desired and interfering both UEs signals arrive at the same angle $\theta_{0}$ of $45^{\circ }$ and when angles of both are mirror reflections of each other such as $\theta_{1}=180^{\circ }-45^{\circ }=135^{\circ }$. The SE expression Equation (17) for NLoS is complex as it consists of special functions and summations. The lower bound for N≥1 is modeled Equation (20).

(20) $$SE_{NLoS}^{0}=E\left\{\log_{2}\left(1+\frac{p{\left|x_{0}\right|}^{2}}{p{\left|x_{1}\right|}^{2}+\sigma^{2}}\right)\right\}\geq \log_{2}\left(1+\frac{N-1}{\bar{g}+u}\right)$$

**Figure 8:**
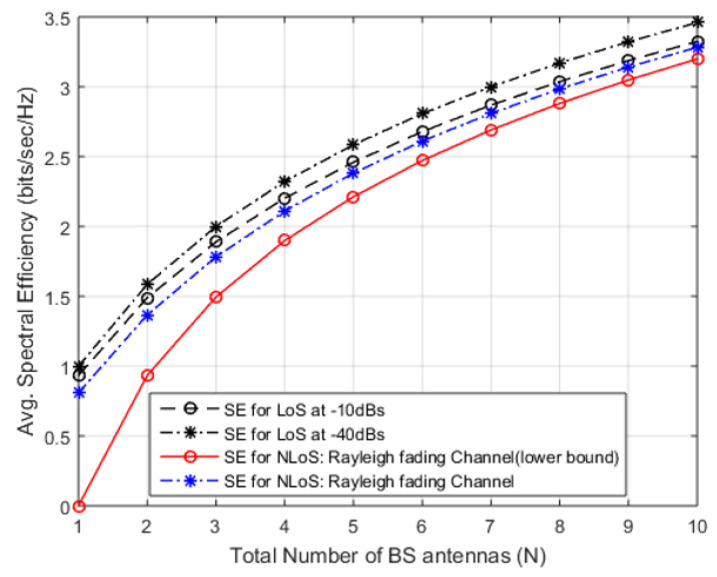
Desired signal, interference from other cell and noise added to the signal during transmission.

The array gain in Equation (17) for calculated for LoS case and NLoS case is calculated in Equation (20) that ended the desired signal-scale as (N−1) instead of *N*. Figure 8 deliberates the LoS cases with N=15,100, and displays the cumulative distribution function at UE angles from 0 to 2π and interference gain. Figure 8 provides an avg. SE realized against the antennas deployed at the BS if the desired user $SNR_{0}$ is considered to be constant 0 dB, $d_{H}$ is fixed at −10 dB and $d_{H}$ is 12. In Figure 8 for LoS from (N=1to10), SE shows rapid improvement from 0.85 to 3.5 bits/s/Hz. This sharp improvement is due to array gain and MR combining. Moreover, after N>10, the SE increases as a monotonic function of *N* that increases as N→∞. Yet again, it is because of MR combining, that gathers extra signal energy (from an array), deprived of amassing energy of interference signal. Figure 8 illustrate the results of LoS scenario at −10 dBs and −40 dBs, in which SE is an increasing function of *N*.

**Figure 9:**
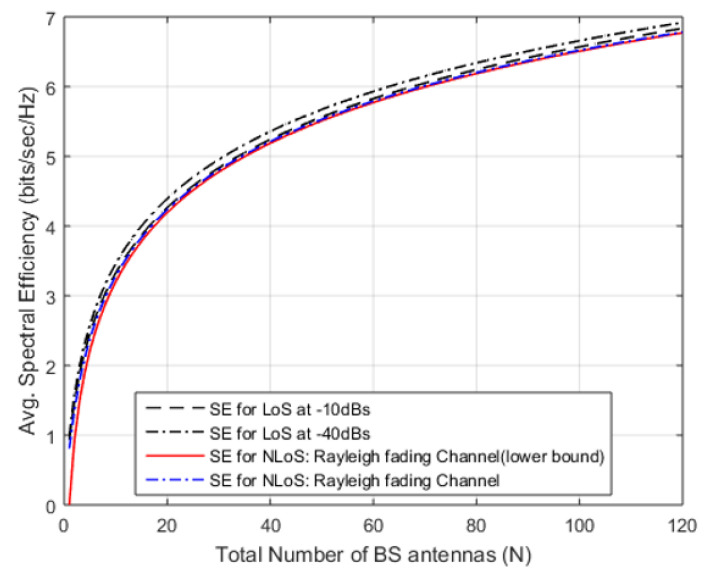
Desired signal, interference from other cell and noise added to the signal during transmission.

Figure 9 shows that there is a slight difference in NLoS and LoS as channel fading puts lesser influence on mutual-information among the signals transmitted and received from extra antennas deployed at BS (N has larger value) [43]. The existing literature [41,44,45,46], on multi-antenna BSs focused on combating channel fading reception focused on combating channel fading, however, our proposal has been attributed with extra DoF and spatial-diversity that spot sovereign fading-realizations. The term channel hardening has been used in [47] to describe a fading channel that behaves almost deterministically due to spatial diversity.

## 4. Conclusions

In this work, the Massive MIMO system was examined for SE augmentation. It concludes that an increased SNR (or more transmit power) increases the SE, however, the constructive effect pushes the system to an interference-limited region that decreases the SE. The proposed mathematical modeling and results show that SE is a linear function of SNR hence, a way of increasing SNR is proposed that provides a constant transmit power and increases cell density. The proposed method provides considerable improvement in SE. Moreover, in channel modeling, an average channel gain has been found inversely proportional to the propagation distance for a fixed path loss coefficient. In this environment, the desired signal power and inter-cell interference upsurge unevenly while D is high. It happens due to the shortened distance between interfering BS and desired BS. Hence, it has been concluded that the interference-limited SE is achievable by increasing cell density but it cannot be sufficiently large in coverage tier. Contrarily, cell densification is a more appropriate method in hotspot tier. Furthermore, it has been observed that area throughput defined in Equation (1) is increased by increasing dell density. The results verify that increasing antennas at BS increases SE without any upper limit while N→∞. It happens because BS has extra DoF and it proficiently processes the received signal through an antenna array. Moreover, it also increases the signal-gain selectively deprived of gathering extra interference but it gathers an extra transmit power. Rebelliously, increasing the transmit power also increases interference. However, SE logarithmically increases *N* (because log2(N)), which does not offer the desired scalability to get improved SE in 5G networks. The proposed model and results show incredible improvements by using ULA configuration in massive MIMO systems however, sub-ULAs would provide better results as compared with ULA configuration while dealing with mmWave MIMO systems. 

## Figures and Tables

**Table 1:** Description of different symbols.

Symbols	Description
$g_{0}$	Average Channel Gain in Cell 0
$g_{1}$	Average Channel Gain in Cell 1
$y_{0}$	Avg. Interference Signal Channel Gain of UEs in Cell 1
$y_{1}$	Avg. Interference Signal Channel Gain of UEs in Cell 0
$\bar{g}\left(0\leq \bar{g}\leq 1\right)$	The ratio of inter and intra cell Gain [14]
$d_{H}$	Antenna Spacing
λ	Wavelength
$m_{0}$	Signal received at Output (UL Communication)
$I_{0}$, $I_{1}$	Information Symbols
$x_{0}$ and $x_{1}$	Channel Responses
$g_{i}$	Large-Scale Fading Coefficient

**Table 2:** Description of simulation symbols and parameter values.

Simulation Symbols	Parameter Values
Antennas in an array (N)	120
The angle of Desired UE $\left(\theta_{0}\right)$	$45^{\circ }$
Range of Angle of Interfering UE $\left(\theta_{1}\right)$	Varies from $\pm 180^{\circ }$
$\bar{g}$	$\left(0\leq \bar{g}\leq 1\right)$
Antenna spacing $\left(d_{H}\right)$	1/2 λ
No. of cells	2
